# Dual Action of Myricetin on *Porphyromonas gingivalis* and the Inflammatory Response of Host Cells: A Promising Therapeutic Molecule for Periodontal Diseases

**DOI:** 10.1371/journal.pone.0131758

**Published:** 2015-06-29

**Authors:** Daniel Grenier, Huangqin Chen, Amel Ben Lagha, Jade Fournier-Larente, Marie-Pierre Morin

**Affiliations:** 1 Oral Ecology Research Group, Faculty of Dentistry, Université Laval, Quebec City, Quebec, Canada; 2 Department of Stomatology, Hubei University of Science and Technology, Xianning City, Hubei Province, China; National University of Singapore, SINGAPORE

## Abstract

Periodontitis that affects the underlying structures of the periodontium, including the alveolar bone, is a multifactorial disease, whose etiology involves interactions between specific bacterial species of the subgingival biofilm and the host immune components. In the present study, we investigated the effects of myricetin, a flavonol largely distributed in fruits and vegetables, on growth and virulence properties of *Porphyromonas gingivalis* as well as on the *P*. *gingivalis*-induced inflammatory response in host cells. Minimal inhibitory concentration values of myricetin against *P*. *gingivalis* were in the range of 62.5 to 125 μg/ml. The iron-chelating activity of myricetin may contribute to the antibacterial activity of this flavonol. Myricetin was found to attenuate the virulence of *P*. *gingivalis* by reducing the expression of genes coding for important virulence factors, including proteinases (*rgpA*, *rgpB*, and *kgp*) and adhesins (*fimA*, *hagA*, and *hagB*). Myricetin dose-dependently prevented NF-κB activation in a monocyte model. Moreover, it inhibited the secretion of IL-6, IL-8 and MMP-3 by *P*. *gingivalis*-stimulated gingival fibroblasts. In conclusion, our study brought clear evidence that the flavonol myricetin exhibits a dual action on the periodontopathogenic bacterium *P*. *gingivalis* and the inflammatory response of host cells. Therefore, myricetin holds promise as a therapeutic agent for the treatment/prevention of periodontitis.

## Introduction

Periodontal disease, which affects the tooth-supporting tissues, is one of most prevalent chronic inflammatory disorder in humans. While mild to moderate forms of the disease affect approximately half of the US adults, up to 15% of the population suffer from severe forms of the disease [1, 2] that will result in a decreased quality of life [3] and high dental care costs [4]. Over the last decade, epidemiologic evidence has accumulated to suggest that periodontal disease represents a risk factor for more serious systemic diseases such as cardiovascular disease, type 2 diabetes and preterm low birth weight [5, 6]. Therefore, it is considered a major public health problem for both developed and developing countries.

Periodontitis that affects the underlying structures of the periodontium, including the alveolar bone, is a multifactorial disease, whose etiology involves interactions between specific bacterial species of the subgingival biofilm and the host immune components [7]. The Gram negative anaerobic bacterium *Porphyromonas gingivalis* is regarded as a key etiological agent involved in the initiation and progression of chronic periodontitis [8]. More specifically, it has been identified in 75% of active sites and in 59.7% of inactive sites in 96% of the patients with progressive adult periodontitis [9]. *P*. *gingivalis* expresses a number of potential virulence factors including fimbriae, cysteine proteinases and lipopolysaccharide (LPS) [10–14]. While fimbriae are considered to be critical factors in adhesion to and invasion of host cells, cysteine proteinases, also known as gingipains, have been suggested to play key roles related to tissue colonization, acquisition of nutrients, evasion of host immune defense, and tissue destruction [10–14]. LPS is a major component of the outer membrane of *P*. *gingivalis* and exhibits a number of biological properties. It is known to induce the secretion of pro-inflammatory mediators and matrix metalloproteinases (MMPs) by mucosal and immune cells [12, 15]. Moreover, the LPS of *P*. *gingivalis* can inhibit alkaline phosphatase activity, collagen type 1 and osteocalcin production, as well as osteoblastic differentiation in human periodontal ligament stem cells [16].

Fibroblasts, the basic gingival tissue cells, contribute to maintaining homeostasis of connective tissue by synthesizing extracellular matrix and collagen, and play a critical role in wound healing [17]. Being a target of LPS through interactions with Toll-like receptors, gingival fibroblasts also play an important role in local inflammatory events through the secretion of inflammatory mediators such as interleukin-6 and interleukin-8 [18]. Interestingly, the regulation of fibroblast inflammatory reactions has been suggested to be one of the ways to prevent/control periodontitis progression [19].

Recently, plant polyphenols have received considerable attention due to their ability to inhibit growth and virulence properties of periodontopathogens and to attenuate the host inflammatory response [20–22]. Such bioactive compounds may be potentially used for adjunctive treatments of refractory and aggressive periodontitis. Myricetin is a naturally occurring flavonol with hydroxyl substitution at the 3, 5, 7, 3’, 4’, and 5’ positions, which is largely distributed in fruits and vegetables. Häkkinen *et al*. [23] analyzed the content of flavonols in 25 edible berries and found that cranberry contained the highest concentration of myricetin (108 mg/kg). Through the years, a number of potential therapeutic properties have been associated to myricetin such as anti-oxidative, antiviral, anti-inflammatory, anti-atherosclerotic and anti-thrombotic activities [24]. The aim of the present study was to investigate the effects of myricetin on growth and virulence properties of *P*. *gingivalis* as well as on the *P*. *gingivalis*-induced inflammatory response in host cells.

## Materials and Methods

### Myricetin

A stock solution of myricetin (Chromadex Inc., Irvine, CA, USA) was prepared in dimethyl sulfoxide (DMSO) at a concentration of 10 mg/ml and kept at -20°C protected from light for up to two months. Preliminary experiments showed that at the dilutions used, the DMSO added had no effects on the assays described below.

### Bacteria and growth conditions

*P*. *gingivalis* (ATCC 33277, ATCC 49417, HW24D1, and W83) was grown in Todd Hewitt broth (BBL Microbiology Systems, Cockeysville, MD, USA) supplemented with 0.001% hemin and 0.0001% vitamin K (THB-HK). Cultures were incubated at 37°C in an anaerobic chamber (N_2_:H_2_:CO_2_ / 75:10:15).

### Minimal inhibitory and minimal bactericidal concentrations

The minimal inhibitory concentrations (MIC) and minimal bactericidal concentrations (MBC) of myricetin were determined by a broth microdilution assay. Briefly, 24-h cultures of *P*. *gingivalis* were diluted in fresh THB-HK to obtain an optical density at 660 nm (OD_660_) of 0.2. Equal volumes (100 μl) of bacteria and serial two-fold dilutions of myricetin (from 500 μg/ml) in culture medium were mixed into the wells of a 96-well microplate. Control wells with no bacteria or no myricetin were also prepared, while doxycycline was used as a reference antibiotic. After an incubation of 24 h at 37°C under anaerobic conditions, bacterial growth was recorded visually. MIC values were determined as the lowest concentrations at which no bacterial growth occurred. To determine the MBC values, aliquots (5 μl) of each well showing no visible growth were spread on sheep blood-supplemented THB agar plates, which were incubated for 3 days at 37°C. The MBC values were determined as the lowest concentrations at which no colony formation occurred. All assays were performed in triplicate to ensure reproducibility.

### Siderophore assay

The universal siderophore assay of Schwyn and Neilands [25] was used to investigate the iron-chelating activity of myricetin. Ferrichrome (Sigma-Aldrich Canada Co., Oakville, ON, Canada), a siderophore produced by *Ustilago sphaerogena* [26] was used as the positive control. All assays were performed in triplicate and the means ± standard deviations were calculated.

### Virulence factor gene expression in *P*. *gingivalis*

The effect of myricetin at sub-MIC on the expression of several *P*. *gingivalis* virulence factor genes involved in host colonization (*fimA*, *hagA*, *hagB*) and tissue destruction (*rgpA*, *rgpB*, *kgp*) was investigated. For this purpose, *P*. *gingivalis* ATCC 33277 was grown to mid-log phase (OD_660_ = 0.45) and then myricetin was added at 100 and 50 μg/ml prior to further incubate at 37°C under anaerobic conditions for 8 h. Control cells were incubated in the absence of myricetin. Bacteria were collected by centrifugation (7,000 x *g* for 5 min) and treated with RNAprotect Bacteria Reagent (Qiagen Canada Inc., Montreal, QC, Canada). Bacterial cells were then lysed and mRNA was isolated and purified using an RNeasy minikit (Qiagen Canada Inc.). mRNA was reverse-transcribed into cDNA prior to performing quantitive PCR analysis for quantification of *fimA*, *hagA*, *hagB*, *rgpA*, *rgpB*, and *kgp* mRNA expression, as described in a previous study [27]. 16S rRNA gene was used as an internal control for data normalization. The primers (Life Technologies Inc., Burlington, ON, Canada) used for the quantitative PCR are listed in Table 1. Three independent experiments were performed in triplicate and a representative set of data (means ± standard deviations) is presented.

**Table 1:** Primers used for the quantitative RT-PCR analysis of virulence factor gene expression in *P*. *gingivalis*.

Gene	Primer sequence	Product size (bp)
16S rRNA	Sense: 5- TGTAGATGACTGATGGTGAAA -3’	138
	Antisense: 5’- ACTGTTAGCAACTACCGATGT -3’	
*fimA*	Sense: 5’- CAGCAGGAAGCCATCAAATC -3’	140
	Antisense: 5’- CAGTCAGTTCAGTTGTCAAT -3’	
*hagA*	Sense: 5’- ACAGCATCAGCCGATATTCC -3’	188
	Antisense: 5’- CGAATTCATTGCCACCTTCT -3’	
*hagB*	Sense: 5’- TGTCGCACGGCAAATATCGCTAAAC -3’	176
	Antisense: 5’- CTGGCTGTCCTCGTCGAAAGCATAC -3’	
*kgp*	Sense: 5’- AGCTGACAAAGGTGGAGACCAAAGG -3’	186
	Antisense: 5’- TGTGGCATGAGTTTTTCGGAACCGT -3’	
*rgpA*	Sense: 5’- GCCGAGATTGTTCTTGAAGC -3’	256
	Antisense: 5’- AGGAGCAGCAATTGCAAAG -3’	
*rgpB*	Sense: 5’- CGCTGATGAAACGAACTTGA-3’	211
	Antisense: 5’- CTTCGAATACCATGCGGTT-3’	

### Type I collagen degradation by *P*. *gingivalis*

A cell-free culture supernatant of *P*. *gingivalis* was obtained from a 24-h culture. Aliquots of supernatant (7.5 μl) were mixed with 127.5 μl of TCNB buffer (50 mM Tris·HCl, 10 mM CaCl_2_, 150 mM NaCl, and 0.05% Brij35, pH 7.5) containing increasing concentrations of myricetin (final concentrations: 0, 25, 50, and 100 μg/ml) and 15 μl of fluorogenic substrate (1 mg/ml; fluorescein-conjugated type I collagen (DQ™ type I collagen; Molecular Probes, Eugene, OR, USA). The assay mixtures were incubated in the dark at room temperature, and the fluorescence was measured after 4 h using a microplate fluorometer (Synergy 2; Bio-Tek Instruments) with the excitation and emission wavelengths set at 490 nm and 520 nm, respectively. All assays were performed in triplicate and the means ± standard deviations were calculated.

### Gelatin degradation by MMP-9

Human recombinant MMP-9 (Calbiochem, San Diego, CA, USA) was diluted to a final concentration of 100 ng/ml in TCNB buffer. The assay mixtures contained 75 μl of MMP-9 at 100 ng/ml, 15 μl of fluorescein-conjugated gelatin (1 mg/ml; DQ™ gelatin; Molecular Probes), 3 μl of myricetin (final concentrations: 0, 25, 50, and 100 μg/ml), and 57 μl of TNCB buffer to make up a final assay volume of 150 μl. The assay mixtures were incubated in the dark for 4 h at 37°C, and the fluorescence was measured using a microplate fluorometer with the excitation and emission wavelengths set at 490 nm and 520 nm, respectively. All assays were performed in triplicate and the means ± standard deviations were calculated.

### *P*. *gingivalis*-induced NF-κB activation

The human monoblastic leukemia cell line U937-3xκB-LUC was kindly provided by Dr. Rune Blomhoff (University of Oslo, Norway). This cell line consists in the U937 cell line stably transfected with a construct containing three NF-κB binding sites from the Ig κ light chain promoter coupled with the gene encoding firefly luciferase (3x-κB-*luc*) [28]. Cells were routinely grown at 37°C in a 5% CO_2_ atmosphere in RPMI-1640 medium (Life Technologies Inc.) supplemented with 10% heat-inactivated fetal bovine serum (FBS), 100 μg/ml penicillin-streptomycin and 75 μg/ml hygromycin B. Preliminary assays using an MTT (3-[4, 5-diethylthiazol-2-yl]-2,5-diphenyltetrazolium bromide) test (Roche Diagnostics, Mannheim, Germany), performed according to the manufacturer’s protocol, revealed that to avoid undesirable effects related to loss of viability of U937-3xκB-LUC, myricetin has to be used at a concentration ≤ 32 μg/ml (S1 Table). The effect of myricetin on NF-κB activation was assessed as follows. Fifty μl of the cell suspension (3 x 10^6^ cells/ml) were seeded on a black bottom, black walls 96-well microplate. Fifty μl of myricetin at non-cytotoxic concentrations were then added. Moreover, an assay was performed using a commercial inhibitor (25 μM; BAY-11-7082; EMD Millipore, Billerica, MA, USA) of the NF-κB signalling pathway. Following a 30-min incubation, 50 μl of *P*. *gingivalis* cells at a multiplicity of infection (MOI) of 100 were added to induce activation of the NF-κB signaling pathway, and the plate was further incubated at 37°C (5% CO_2_) for 6 h. NF-κB activation was then monitored using a luciferase assay kit (Bright-Glo Luciferase Assay System; Promega, Madison, WI, USA) by adding 100 μl of luciferase substrate to the wells at room temperature. Luminescence was recorded using the luminometer option of a microplate reader (Synergy 2) within 2 min after substrate addition. Two independent experiments were performed in triplicate and a representative set of data (means ± standard deviations) is presented.

### IL-6, IL-8, and MMP-3 secretion by *P*. *gingivalis*-stimulated gingival fibroblasts

The primary human gingival fibroblast cell line HGF-1 (ATCC CRL-2014; American Type Culture Collection, Manassas, VA, USA) was cultured in Dulbecco’s modified Eagle’s medium (DMEM; HyClone Laboratories, Logan, UT, USA) supplemented with 4 mM L-glutamine, 15% FBS, and 100 μg/ml of penicillin G-streptomycin at 37°C in a 5% CO2 atmosphere. All experiments were performed using the same lot of cells. Preliminary assays using an MTT test revealed that to avoid undesirable effects related to loss of viability of fibroblasts, myricetin has to be used at a concentration ≤ 100 μg/ml (S2 Table). The effect of myricetin on IL-6, IL-8, and MMP-3 secretion by *P*. *gingivalis*-stimulated fibroblasts was assessed as follows. Cells were cultivated to confluence into 12-wells tissue culture plate and treated with myricetin at non-cytotoxic concentrations for 2 h at 37°C in 5% CO_2_ prior to stimulation with *P*. *gingivalis* cells (MOI = 100) or LPS (1 μg/ml). After 24 h of incubation, conditioned media were removed and stored at -20°C until use. Cells incubated in culture medium with or without myricetin but not stimulated with bacteria or LPS were used as controls. Commercial enzyme-linked immunosorbent assay (ELISA) kits (eBioscience Inc., San Diego, CA, USA) were used to quantify IL-6, IL-8, and MMP-3 in the conditioned media according to the manufacturer’s protocols. Two independent experiments were performed in triplicate and a representative set of data (means ± standard deviations) is presented.

### Statistics

Unless specified otherwise, all assays were performed in triplicate and the means ± standard deviations were calculated. Differences between means were analyzed for statistical significance using the Student’s t-test and were considered significant at *P* < 0.01 or *P* < 0.05.

## Results

The antibacterial activity of myricetin was determined against four strains of *P*. *gingivalis*. As reported in Table 2, the MIC values of myricetin were in the range of 62.5 to 125 μg/ml, while the MBC values were in the range of 125 to 250 μg/ml. The reference antibiotic doxycycline, which is commonly used in adjunctive periodontal therapy, showed lower values of MIC (0.78–1.56 μg/ml) and MBC (12.5–50 μg/ml).

**Table 2:** Minimal inhibitory concentration (MIC) and minimal bactericidal concentration (MBC) values of myricetin and doxycycline against four strains of *P*. *gingivalis*.

	Myricetin		Doxycycline	
Strain	MIC^1^	MBC^2^	MIC	MBC
ATCC 33277	125	250	0.78	12.5
ATCC 49417	125	250	1.56	50
HW24D1	62.5	125	0.78	12.5
W83	62.5	125	0.78	12.5

^1^ MIC values in μg/ml

^2^ MBC values in μg/ml

**Fig 1:**
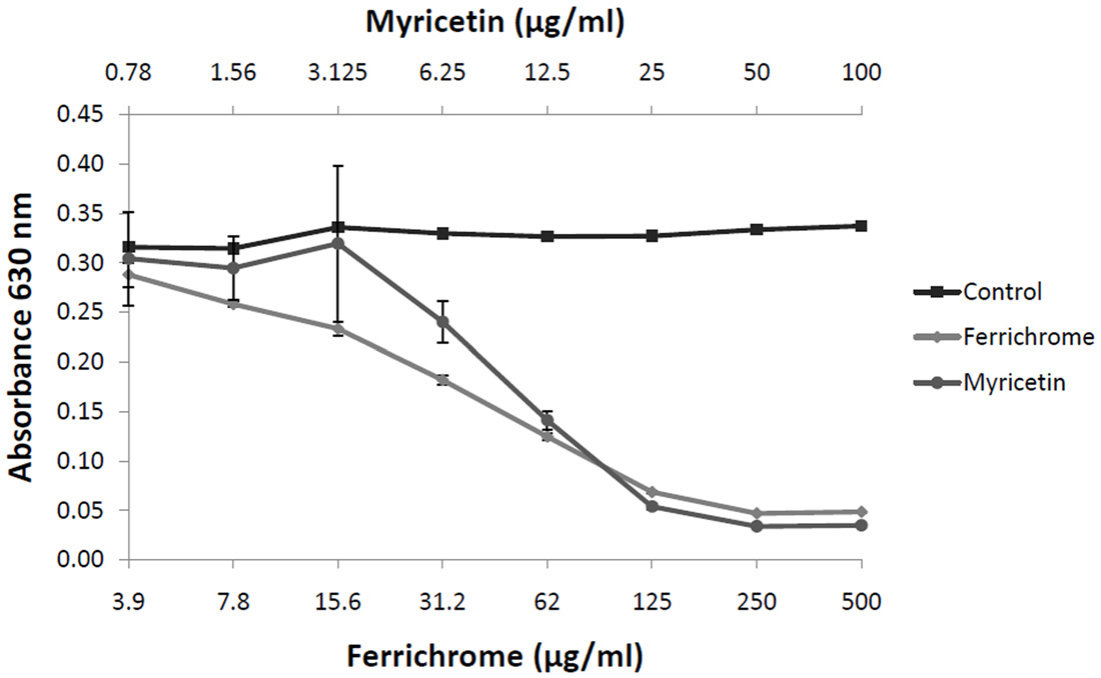
Iron-chelating activity of myricetin as determined by the universal siderophore colorimetric assay using chrome azurol sulfate. Reduction of A_630_ occurs when a strong chelator removes the iron from the dye chrome azurol sulfate. Ferrichrome, a siderophore produced by *U*. *sphaerogena*, was used as positive control.

A preliminary assay revealed that myricetin did not possess membrane permeabilizing activity on *P*. *gingivalis* (data not shown) that may have been involved in its antibacterial property. In an attempt to identify the mechanism by which myricetin exhibits antibacterial activity against *P*. *gingivalis*, we investigated whether this flavonol possesses a siderophore activity resulting in the chelation of iron, an essential element for most bacteria. Data presented in Fig 1 indicate that myricetin dose-dependently chelates iron in a universal siderophore assay using chrome azurol sulfate. The activity reached a maximum at a concentration of myricetin ≥ 50 μg/ml. The iron-chelating activity of myricetin was stronger than that observed with ferrichrome, a reference siderophore produced by *U*. *sphaerogena*.

**Fig 2:**
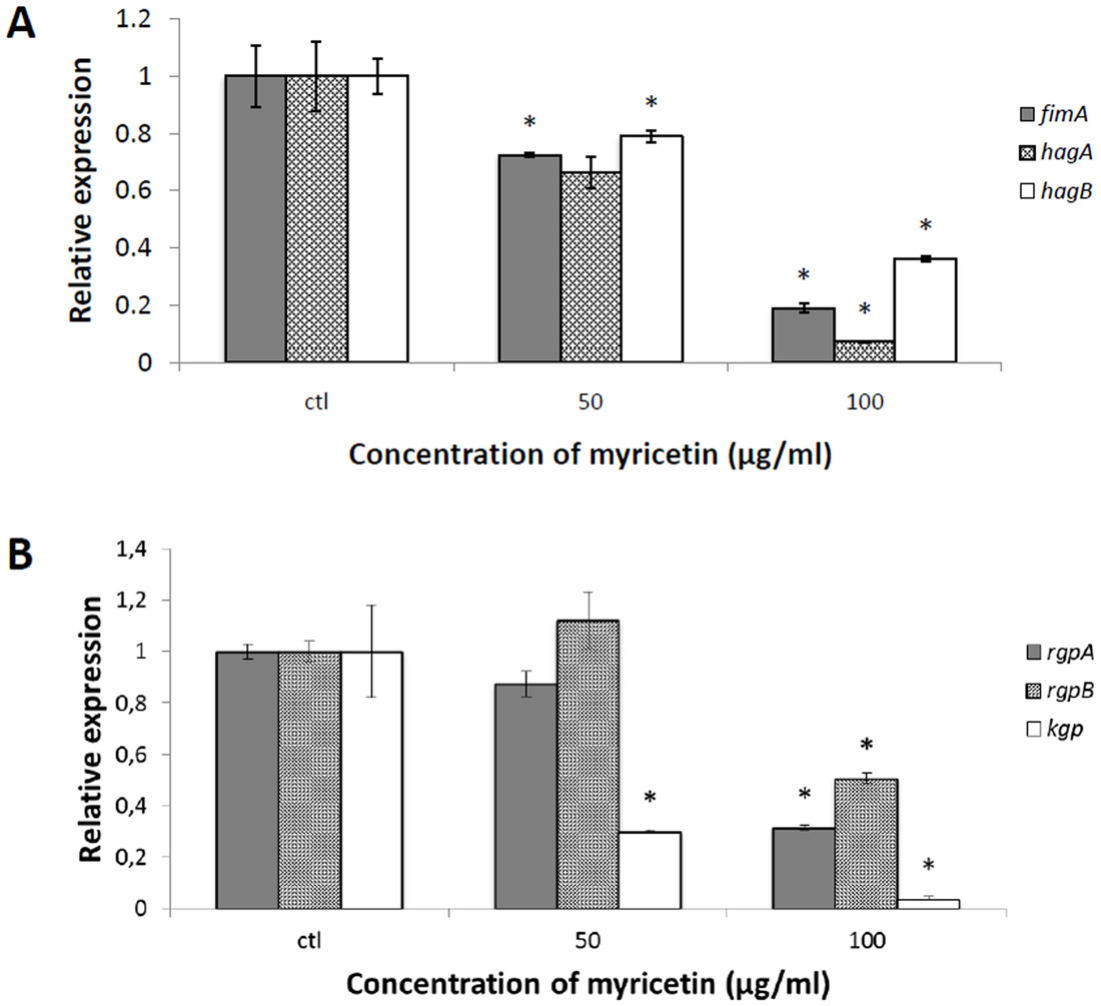
Effect of myricetin on mRNA expression of *fimA*, *hagA*, and *hagB* (Panel A), and *rgpA*, *rgpB*, and *kgp* (Panel B) genes in *P*. *gingivalis* ATCC 33277. Bacteria were incubated (8 h; anaerobic condition) in the presence of myricetin (50 and 100 μg/ml). Data are expressed as means ± standard deviations. The expression was normalized to 16S rRNA. *, significantly different (*P* < 0.01) compared to untreated control.

We then investigated the effect of myricetin on the expression of several virulence factor genes by *P*. *gingivalis*, relative to the expression of 16S rRNA. With this purpose, an early exponential growth phase culture of *P*. *gingivalis* (ATCC 33277) was exposed for 8 h under anaerobic conditions to myricetin at two sub-MICs (50 and 100 μg/ml) prior to monitoring mRNA expression by quantitative RT-PCR. Fig 2A reports the data for three genes (*fimA*, *hagA*, *hagB*) involved in bacterial colonization. A significant (at *P* < 0.01) decrease in the expression of all genes was observed following exposure of *P*. *gingivalis* to myricetin at a concentration of 100 μg/ml, while at 50 μg/ml, only *fimA* and *hagB* were significantly decreased. More specifically, myricetin at 100 μg/ml caused a decrease of 81, 93, and 64% for *fimA*, *hagA*, and *hagB*, respectively. When exposed to myricetin, the expression of *rgpA*, *rgpB*, and *kgp*, three cysteine protease genes related to inactivation of host defense mechanisms, tissue destruction, and nutrient acquisition, was also significantly down-regulated. At 100 μg/ml, the expression of *rgpA*, *rgpB*, and *kgp* decreased by 68, 49, and 96%, respectively (Fig 2B).

We then evaluated the ability of myricetin to inhibit the degradation of type I collagen by the collagenase activity found in a culture supernatant of *P*. *gingivalis*. Data presented in Table 3 showed that this flavonol caused a dose-dependent inhibition of *P*. *gingivalis* collagenase activity. At 100 μg/ml, an almost complete inhibition (95 ± 12%) of type I collagen was obtained. The proteinase inhibitory activity of myricetin was also tested on MMP-9, a cation-dependent endopeptidase secreted by several human cell types. As for the collagenase activity of *P*. *gingivalis*, a dose-dependent inhibition by myricetin was observed. At 100 μg/ml, myricetin caused an inhibition of 91% of MMP-9 activity.

**Table 3:** Effects of myricetin on the activity of *P*. *gingivalis* collagenase and human MMP-9.

	Inhibition (%)	
Concentration of myricetin (μg/ml)	P. gingivalis collagenase	MMP-9
0	0	0
5	61 ± 9*	14 ± 6
25	78 ± 14*	43 ± 15*
100	95 ± 12*	91 ± 9*

*, Significantly different (*P* < 0.01) from control (absence of myricetin)

**Fig 3:**
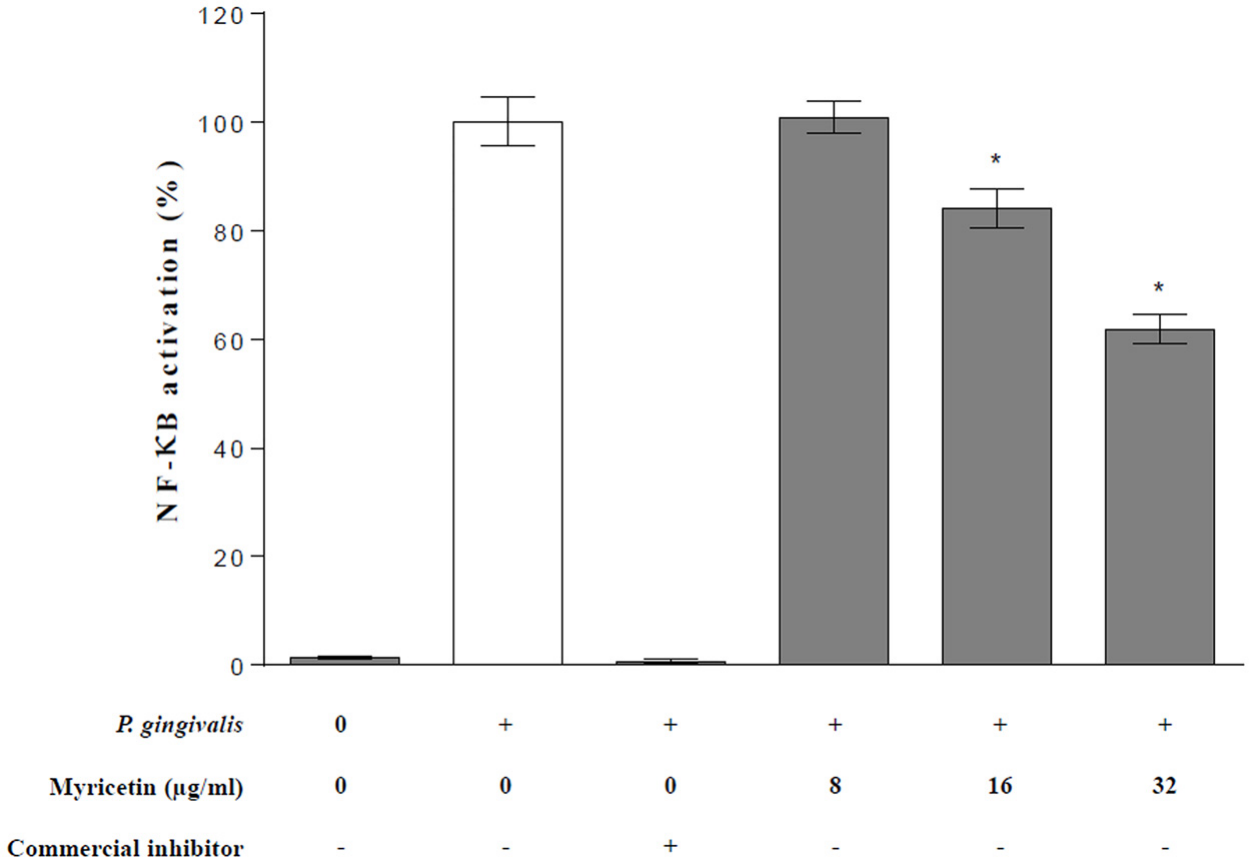
Effect of myricetin on *P*. *gingivalis*-induced NF-κB activation using the U937-3xκB-LUC cell line model. Cells were treated for 30 min with myricetin prior to stimulation with *P*. *gingivalis* (ATCC 33277) at MOI of 100. A value of 100% was assigned to NF-κB activation induced by *P*. *gingivalis*. The commercial inhibitor BAY-11-7082 was used as positive inhibitory control. *, significant decrease at *P* < 0.05 compared to *P*. *gingivalis*-stimulated monocytes not treated with myricetin.

To investigate the anti-inflammatory properties of myricetin, the U937-3xκB-LUC cell line transfected with a luciferase reporter gene was used to determine the effect of this flavonol on *P*. *gingivalis*-mediated activation of the NF-κB signaling pathway. Fig 3 shows that *P*. *gingivalis* induced NF-κB activation, while the commercial inhibitor (BAY-11-7082) completely prevented this activation. Myricetin dose-dependently inhibited the *P*. *gingivalis*-induced NF-κB activation. More specifically, myricetin at 16 and 32 μg/ml significantly decreased the NF-κB activation by 15.9% and 38.2%, respectively.

**Fig 4:**
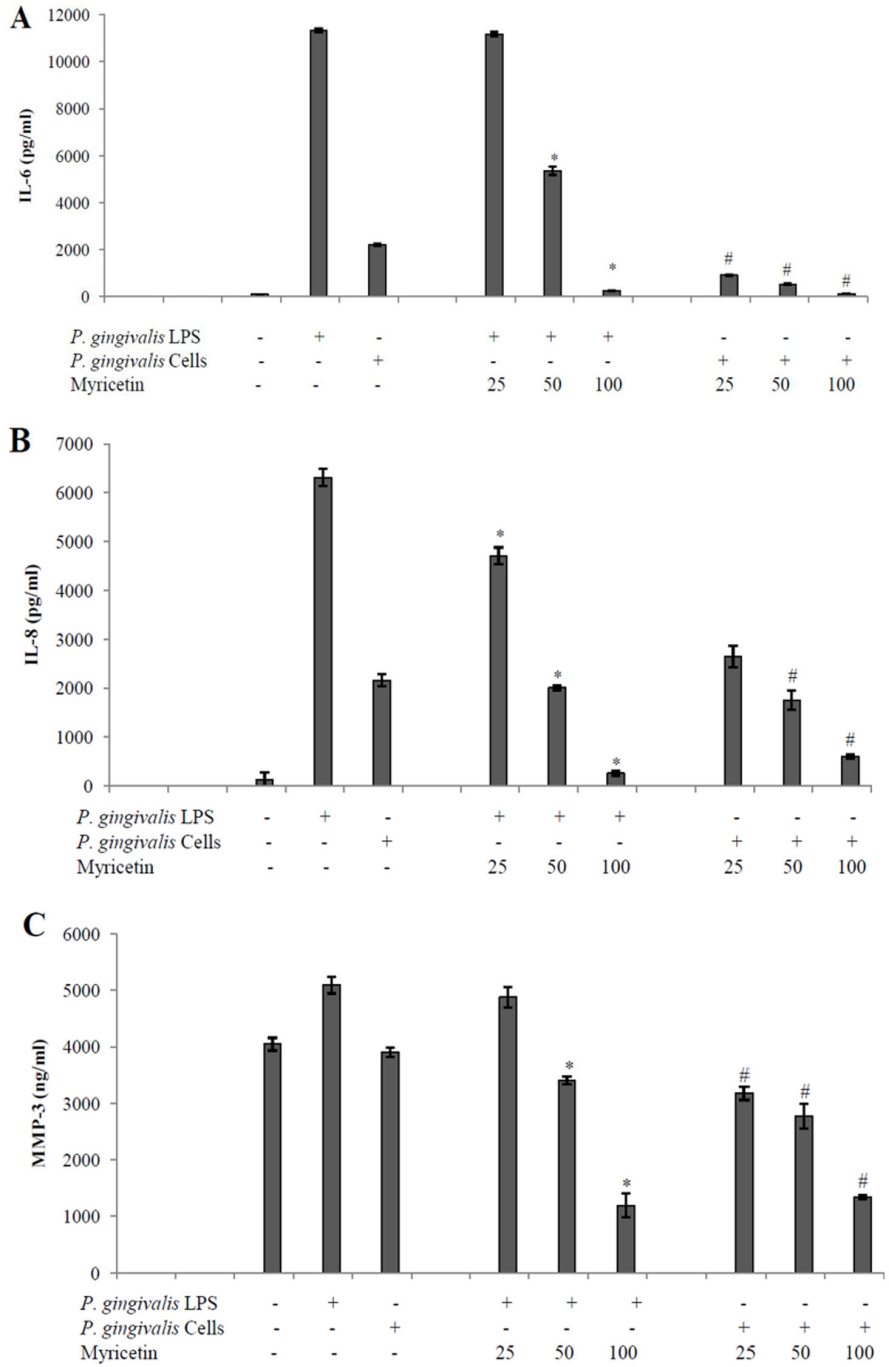
Effect of myricetin on the secretion of IL-6 (Panel A), IL-8 (Panel B), and MMP-3 (Panel C) by human gingival fibroblasts stimulated with *P*. *gingivalis* (ATCC 33277) whole cells (MOI of 100) or LPS (1 μg/ml). Gingival fibroblasts were treated for 2 h with myricetin prior to stimulation with *P*. *gingivalis* for 24 h. *, significant decrease at *P* < 0.05 compared to *P*. *gingivalis*-stimulated gingival fibroblasts not treated with myricetin. #, significant decrease at *P* < 0.05 compared to LPS-stimulated gingival fibroblasts not treated with myricetin.

To further support that myricetin possesses an anti-inflammatory activity, we then examined the effect of this flavonol on the secretion of IL-6, IL-8 and MMP-3 by *P*. *gingivalis*-stimulated human gingival fibroblasts. As expected, *P*. *gingivalis* LPS and to a lesser extent whole bacteria markedly increased the secretion of IL-6 and IL-8 (Fig 4A and 4B). The secretion of MMP-3 by gingival fibroblasts was only induced by *P*. *gingivalis* LPS (Fig 4C). Myricetin dose-dependently attenuated *P*. *gingivalis* LPS- and whole bacteria-induced IL-6, IL-8 and MMP-3 secretion (Fig 4). At a concentration of 50 μg/ml, myricetin inhibited by 53% and 76% the secretion of IL-6 by *P*. *gingivalis* LPS- and whole bacteria-stimulated gingival fibroblasts, respectively. Regarding IL-8, myricetin at 50 μg/ml was more effective in inhibiting LPS-induced (68% inhibition) than whole bacteria-induced (19% inhibition) secretion. Lastly, the secretion of MMP-3 by *P*. *gingivalis* LPS- and whole bacteria-stimulated gingival fibroblasts was markedly reduced by myricetin. At 50 and 100 μg/ml of myricetin, the amounts of MMP-3 secreted were below the basal levels (no *P*. *gingivalis* stimulation).

## Discussion

Scientific evidence showed that conventional periodontal treatment involving scaling and root planning procedures is highly effective to resolve most cases of periodontitis. However, there are still some situations where satisfactory clinical outcomes in periodontitis patients cannot be obtained. In such cases, adjunctive therapies may be appropriate, more specifically the use of bioactive agents possessing an antimicrobial activity. Over the years, research carried out by various groups has identified a number of human health benefits associated with flavonols such as myricetin (3, 5, 7, 3’, 4’, 5’ hexahydroxyflavone) [24, 29]. In this study, we investigated the effects of myricetin on growth and virulence properties of *P*. *gingivalis* as well as on the inflammatory response induced by *P*. *gingivalis* in two human cell types (monocytes and fibroblasts).

We first showed that myricetin was active against *P*. *gingivalis* with MIC values in the range of 62.5 to 125 μg/ml. In a previous study, Cai and Wu [30] also reported a growth inhibitory activity of myricetin against *P*. *gingivalis*, while this flavonol was poorly active on the two Gram positive bacteria tested (*Streptococcus mutans*, *Actinomyces viscosus*). Moreover, using an agar diffusion method, Puupponen-Pimiä et al. [31] compared different flavonols (quercetin, rutin, kaempferol, myricetin) and showed that myricetin possesses the strongest antimicrobial activity as well as the largest inhibitory spectrum. These authors suggested that the high degree of hydroxylation of myricetin was associated with the marked antimicrobial property of the molecule. In an attempt to identify the mechanism by which myricetin may inhibit growth of *P*. *gingivalis*, we tested its ability to chelate iron, which is an essential nutrient for most bacteria and consequently an important factor during the course of infections [32, 33]. Using a universal siderophore assay, we demonstrated that myricetin possesses a strong iron-chelating activity that may contribute to the antibacterial activity of this flavonol. Iron chelators have been previously reported to exert antibacterial properties. More specifically, deferasirox and 2,2’-dipyridyl were found to inhibit the growth of *Prevotella intermedia* [34] and *P*. *gingivalis* [35], respectively. Interestingly, the iron-chelating activity of myricetin could also regulate MMP activity by chelating iron and other transition metals present in trace amounts in inflamed periodontal tissues. In fact, the generation of reactive oxygen species, which activate MMPs that contribute to tissue breakdown, may be prevented by this chelating activity [36]. The ability of myricetin to inhibit DNA synthesis may also contribute to growth inhibition of *P*. *gingivalis*, as previously reported for *Staphylococcus aureus* [37].

We then explored the ability of myricetin to attenuate the virulence of *P*. *gingivalis* by reducing the expression of genes coding for important virulence factors. Myricetin at sub-inhibitory concentrations was found to significantly inhibit the expression of *fimA*, *hagA*, and *hagB*, which are involved in bacterial colonization [38, 39]. Moreover, the expression of *rgpA*, *rgpB*, and *kgp*, three protease genes related to inactivation of host defense mechanisms, tissue destruction, and nutrient acquisition [11], was also down-regulated by myricetin. The ability of plant polyphenols to regulate gene expression has been previously reported. More specifically, our laboratory showed that the anthraquinone rhein from rhubarb also decreases the expression of important virulence factor genes in *P*. *gingivalis* [27]. It can be hypothesized that the mechanism by which myricetin down-regulates virulence factor gene expression in *P*. *gingivalis* involves its iron-chelating activity. This is supported by previous studies [40, 41] reporting that iron-depleted conditions can either up- or down-regulate the expression of a large array of genes in *P*. *gingivalis*.

Based on our knowledge of the mechanisms of host- and bacteria-mediated periodontal tissue destruction, targeting proteinases is considered as a valuable strategy for periodontal treatment. In this regard, a number of authors brought evidence that periodontitis progression can be hampered by successfully inhibiting both bacteria- and host-derived proteinases involved in connective tissue destruction of the periodontium [42, 43]. We showed that myricetin was effective in inhibiting collagen degradation mediated by the cysteine proteinase gingipains secreted by *P*. *gingivalis*. This is in agreement with the study of Jin et al. [44] who reported that myricetin can act as a dual inhibitor of the cysteine proteinase falcipain-2 and plasmepsin II produced by *Plasmodium falciparum*. This anti-proteinase activity of myricetin has been suggested to contribute to the antimalarial activity of this flavonol. Moreover, our data also showed that myricetin was highly effective in inhibiting the gelatinase activity of MMP-9. MMPs are endopeptidases that contain a zinc ion in their active site and for which an over-production, such as observed during periodontitis, contributes to tissue destruction [45]. Interestingly, Wei and Guo recently reported that myricetin chelates zinc at the 3-hydroxyl-4-keto site [46]. This property of myricetin is likely involved in the capacity of this flavonol to inhibit MMP-9.

Therapeutic approaches that inhibit inflammatory mediator production represent a promising alternative for controlling inflammatory diseases such as periodontal diseases [47]. Given that the transcription factor NF-κB controls the expression of a large array of genes involved in inflammation [48], we used the human monoblastic leukemia cell line U937-3xκB-LUC to evaluate the ability of myricetin to inhibit activation of the NF-κB signaling pathway in view to support the potential of this flavovol to reduce the host inflammatory response associated with periodontal diseases. Myricetin was found to dose-dependently prevent NF-κB activation induced by *P*. *gingivalis* LPS and whole bacteria in this cell line, although this property should also be tested using primary cells. The iron-chelating property of myricetin may contribute to this inhibition since iron chelators have been previously reported to inhibit the NF-κB signalling pathway in various cell types [49, 50].

Excessive inflammatory response to bacterial challenge resulting in IL-6 and IL-8 secretion play a key role in periodontal tissue destruction. IL-6 is a pleiotropic cytokine with a broad range of humoral and cellular immune effects related to inflammation, host defense, and tissue injury [51]. Moreover, IL-6 has also been shown to mediate activation and differentiation of B and T cells, macrophages, osteoclasts and chondrocytes [52]. IL-8 is a potent chemoattractant and activator for neutrophils, which plays a crucial role in the first line of host defense against microorganisms [53]. To confirm the anti-inflammatory property of myricetin, we used a gingival fibroblasts stimulated with either whole bacteria or LPS of *P*. *gingivalis* and monitored IL-6 and IL-8 secretion. Our results showed that bacterial cells and LPS induced the secretion of high amounts of IL-6 and IL-8 that may be attenuated by the presence of myricetin. The inhibitory effect of myricetin on the secretion of both cytokines was concentration-dependent. The above data supporting the anti-inflammatory potential of myricetin are in agreement with previous studies that showed that this flavonol can inhibit the expression of additional inflammatory mediators by lipoteichoic acid-stimulated gingival fibroblasts, including interleukin-1β and cyclooxygenase-2 [54].

Gingival fibroblasts are the most abundant resident cells in periodontal tissue and can be considered as a major source of MMPs into periodontal diseased tissue [55]. MMP-3 is a broad spectrum MMP and has a pivotal role in activating latent MMPs including pro-MMP-1, -8 and -9 [56]. Consequently, inhibition of MMP-3 secretion by gingival fibroblasts may reduce periodontal tissue degradation. *P*. *gingivalis* LPS, but not whole bacteria, markedly increased the production of MMP-3 by fibroblasts. The inhibition pattern of MMP-3 following stimulation with *P*. *gingivalis* cells or LPS in the presence of myricetin was similar as that of IL-6 and IL-8.

## Conclusion

Our study brought clear evidence that the flavonol myricetin exhibits a dual action on the periodontopathogenic bacterium *P*. *gingivalis* and the inflammatory response of host cells. On the one hand, myricetin inhibits growth, virulence factor gene expression, and collagenase activity of *P*. *gingivalis*. On the other hand, it also blocks the activation of the NF-κB signaling pathway in monocytes as well as the secretion of cytokines (IL-6, IL-8) and MMP-3 by gingival fibroblasts. By acting on several targets related to the etiology of periodontitis, myricetin holds promise as a therapeutic agent for treating/preventing these diseases. While effective concentrations of myricetin may be difficult to reach through the ingestion of myricetin-containing food, this bioactive molecule could be applied locally to diseased periodontal sites by irrigation or by the insertion of resorbable fibers to reduce the growth and virulence properties of *P*. *gingivalis* as well as the inflammation process.

## Supporting Information

S1 Table Effects of myricetin, in the absence or presence of *P*. *gingivalis* cells, on the viability of U937-3xκB-LUC cell line, as determined with a MTT assay. (DOC)

S2 Table Effects of myricetin, in the absence or presence of *P*. *gingivalis* cells or LPS, on the viability of the human gingival fibroblast HGF-1 cell line, as determined with a MTT assay. (DOC)
