# Supplementary material for: Dual Action of Myricetin on Porphyromonas gingivalis and the Inflammatory Response of Host Cells: A Promising Therapeutic Molecule for Periodontal Diseases
Source: PLoS One. 2015 Jun 29;10(6):e0131758. doi: 10.1371/journal.pone.0131758 (PMC4487256; doi:10.1371/journal.pone.0131758)
Supplement: S1 Table — (DOC) [file pone.0131758.s001.doc]

S1 Table. Effects of myricetin, in the absence or presence of *P. gingivalis* cells, on the viability of U937-3xκB-LUC cell line, as determined with a MTT assay.

Treatment Cell viability (%)

None 100 ± 8

*P. gingivalis* (MOI = 100) 91 ± 5

Myricetin (64 µg/ml) 76 ± 11

Myricetin (64 µg/ml) + *P. gingivalis* (MOI = 100) 71 ± 14

Myricetin (32 µg/ml) 88 ± 9

Myricetin (32 µg/ml) + *P. gingivalis* (MOI = 100) 85 ± 6

Myricetin (16 µg/ml) 92 ± 11

Myricetin (16 µg/ml) + *P. gingivalis* (MOI = 100) 95 ± 3
