# Supplementary material for: Dual Action of Myricetin on Porphyromonas gingivalis and the Inflammatory Response of Host Cells: A Promising Therapeutic Molecule for Periodontal Diseases
Source: PLoS One. 2015 Jun 29;10(6):e0131758. doi: 10.1371/journal.pone.0131758 (PMC4487256; doi:10.1371/journal.pone.0131758)
Supplement: S2 Table — (DOC) [file pone.0131758.s002.doc]

S2 Table. Effects of myricetin, in the absence or presence of *P. gingivalis* cells or LPS, on the viability of the human gingival fibroblast HGF-1 cell line, as determined with a MTT assay.

Treatment Cell viability (%)

None 100 ± 11

*P. gingivalis* (MOI = 100) 95 ± 6

*P. gingivalis* (LPS = 1 µg/ml) 103 ± 2

Myricetin (200 µg/ml) 71 ± 13

Myricetin (200 µg/ml) + *P. gingivalis* (MOI = 100) 63 ± 10

Myricetin (200 µg/ml) + *P. gingivalis* (LPS = 1 µg/ml) 73 ± 8

Myricetin (100 µg/ml) 89 ± 12

Myricetin (100 µg/ml) + *P. gingivalis* (MOI = 100) 83 ± 8

Myricetin (200 µg/ml) + *P. gingivalis* (LPS = 1 µg/ml) 87 ± 5

Myricetin (50 µg/ml) 92 ± 3

Myricetin (50 µg/ml) + *P. gingivalis* (MOI = 100) 92 ± 7

Myricetin (200 µg/ml) + *P. gingivalis* (LPS = 1 µg/ml) 98 ± 2
